# Directionality in hyperbrain networks discriminates between leaders and followers in guitar duets

**DOI:** 10.3389/fnhum.2013.00234

**Published:** 2013-06-04

**Authors:** Johanna Sänger, Viktor Müller, Ulman Lindenberger

**Affiliations:** Center for Lifespan Psychology, Max Planck Institute for Human DevelopmentBerlin, Germany

**Keywords:** interbrain connectivity, EEG hyperscanning, oscillatory synchronization, social cognition, action coordination, music, graph analysis

## Abstract

To investigate whether directionality in hyperbrain networks reflects different roles during interpersonal action coordination (IAC), we recorded EEG data from pairs of guitarists playing together as musical leaders versus followers. We used an asymmetric index of in-phase synchronization to analyze hyperbrain networks of directed functional connectivity in the alpha and beta frequency ranges for time segments around coordinated play onsets. After exploring the small-world characteristics of the networks at different thresholds, we examined the directed connection strengths within and between brains. As predicted, we found evidence suggesting that the musical roles of leader and follower are associated with different patterns of directed between-brain couplings. The functional significance of these differences for IAC requires further study.

## Introduction

Social-cognitive neuroscience has begun to investigate interbrain synchronization during interpersonally coordinated actions (Hari and Salmelin, 1997; Sänger et al., 2011; Konvalinka and Roepstorff, 2012). Such synchronization phenomena are assumed to relate to the interacting agents' representations of their own and their partner's actions. These representations are presumably formed to achieve temporally aligned activity modulation (Lindenberger et al., 2009; Sänger et al., 2011). A particularly clear example is represented by ensemble musicians, who presumably achieve and maintain synchrony by emulating each other's concurrent and predicting each others' future actions (Rasch, 1979; Keller et al., 2007).

In line with these considerations, our prior research with guitar duets showed that: (1) musical coordination points are accompanied by increased phase locking within and between the guitarists' brains (Lindenberger et al., 2009; Sänger et al., 2012); (2) hyperbrain networks show small-world properties (Sänger et al., 2012); (3) the musical roles of leader and follower are associated with differences in within- and between-brain synchronization patterns (Sänger et al., 2012). Here, we reanalyze data originally reported in one of these studies (i.e., Sänger et al., 2012) to examine whether leader and follower differ in the directionality of between-brain couplings at musical coordination points.

Real-world social interactions often require role differentiation among interaction partners, such as “initiator” and “responder” (cf. Schilbach et al., in press). To implement this distinction, Sänger et al. (2012) specified the musical roles of leader and follower in the context of a guitar duet. The leader was asked to coordinate play onsets and set the tempo, and the follower was instructed to play in accordance to the leader's specifications. The two musicians played different but equally important voices (for details, see Sänger et al., 2012). In our original report, we observed asymmetric patterns of phase locking at single electrodes, which reflected differences in musical roles. Here, we complement these earlier analyses by applying a single-trial measure of time-lagged phase coupling, that is, the Integrative Coupling Index (ICI) as described in Müller and Lindenberger (2011). This entails two major advancements: First, this measure directly specifies the direction of functional connectivity between the two brains, which was not possible with the symmetric index of phase coherence we formerly used and might give information on asymmetric between-brain synchronization patterns in association with the different musical roles. Second, this measure captures phase coupling across time as opposed to phase locking respectively coherence across trials, which were captured by our former analyses. With this, we come closer to describing real-time synchronization during interpersonal action coordination (IAC) in guitar duets.

Only a few studies have examined directed functional connectivity among multiple brains so far. Astolfi et al. (2010) investigated groups of four card players who played in teams of two against two. Functional connectivity was found among the signals of players from the same team, suggesting that the players' brain activity was functionally coupled only if they were motivated to coordinate their behavior. The direction of connectivity went from the first player, who opened the team's move by choosing one card, to the second player of the same team, who had to select another card to exploit the situation set by the first player's choice.

The roles of sender and receiver in interpersonal communication were also examined with magnetic resonance (MR) imaging. For example, Anders et al. (2010) investigated the facial communication of affect. Senders were asked to indulge in and accordingly express different emotional states. Receivers were supposed to empathize with the senders when watching video recordings of the senders' facial expressions. Using this procedure, neural activity in a given perceiver's brain could be predicted from activity in the sender's brain. Interestingly, the temporal delay in the activity of the receiver's brain relative to the activity of the sender's brain decreased over time, which, according to the authors, may reflect a “tuning in” of the receiver with the sender.

Schippers et al. (2010) investigated gestural communication by scanning senders while gesturing a given word, and subsequently scanning receivers while trying to guess the word from the recording of the sender's gestures. They found that the moment-to-moment activity in the receiver's putative mirror neuron system (pMNS) mirrored the recent past of the activity in the sender's pMNS. The association between the two brains was significantly reduced when the receiver passively watched the video rather than actually trying to guess the word. Similarly, Stephens et al. (2010) showed that time-shifted couplings between speakers and listeners in brain areas involved in linguistic, semantic, and social processing were contingent upon communication success. The speaker's brain activity preceded the listener's activity in posterior areas, including the right temporal-parietal junction and the precuneus. However, in the striatum and in anterior frontal areas, including medial and dorsolateral prefrontal cortex, the listener's brain activity actually preceded the speaker's brain activity. The extent of cortical areas in which the listeners' activity preceded the speaker's was strongly related to story comprehension, presumably reflecting prediction activity on the part of the receiver (Stephens et al., 2010).

It is worth noting that some of the prior findings, especially when based on MR imaging, may reflect the more general process of mentalizing, or representing another person's mental states, rather than more specific processes related to joint action. Following Sebanz et al. (2006), we define joint action as a type of interaction in which individuals coordinate their actions in space and time to bring about a change in the environment. Musical production of the kind considered here clearly qualifies as joint action, as the individual players work on a joint product (e.g., the interpretation of a piece of music). Hence, the paradigm of observing musicians interpreting a piece of music is well suited to examine empirically whether directionality in interbrain couplings is associated with the interaction partners' contributions to the joint action.

We expected a complex pattern of directed oscillatory couplings within and between brains. With regard to frequencies, we restricted our analyses to the alpha and beta bands. On the one hand, these have been found to be functionally relevant for joint action in earlier studies (Babiloni et al., 2007; Tognoli et al., 2007; Astolfi et al., 2010; Dumas et al., 2010). On the other hand, alpha and beta have been associated with mirroring processes (Tremblay et al., 2004) and finger movements (Hari and Salmelin, 1997), both of which are considered crucial for guitar duet playing. In line with Sänger et al. (2012), we concentrated our efforts on the time segments around play onsets, which we considered to be points of high coordinative demand. We hypothesized that the musical roles of leader and follower would be associated with asymmetric patterns of between-brain functional connectivity. This asymmetry should not be attributable to differences in motor output or sensory input, as these did not differ systematically between the two players. Rather, the asymmetric coupling should refer to differences in oscillatory activity associated with the two musical roles. Specifically, we expected to find a dominance of functional connectivity directed from the leader to the follower, based on the notion that the leading role should be associated to an anteriority in action representation.

## Materials and methods

### Research participants

We tested 16 non-overlapping couples of guitarists. Four of these were excluded because they provided fewer than 30 artifact-free trials. Of the remaining 12 duets, seven were male, four were mixed, and only one duet consisted of two female players. All except one guitarist were right-handed. The participants were aged 20–58 years (*M* = 35.58, *SD* = 1.82) and had been playing guitar for 22.92 years on average (*SD* = 11.64). All participants except two played the guitar more than once a week. All guitarists were currently or had formerly been playing in a musical ensemble. Ten had studied or were studying music at a conservatory. An attempt was made to match the duet partners with respect to age and playing experience (for details, see **Appendix A**). Potential differences between the duets with regard to musical proficiency cannot be denied, but are assumed to range on such a high level that they should not compromise our results. All individuals volunteered for participation in the experiment, and gave their written informed consent prior to their inclusion in the study. The Ethics Committee of the Max Planck Institute for Human Development approved the study. The study was performed in accordance with the ethical standards laid down in the 1964 Declaration of Helsinki.

### Experimental procedure

**Figure 1:**
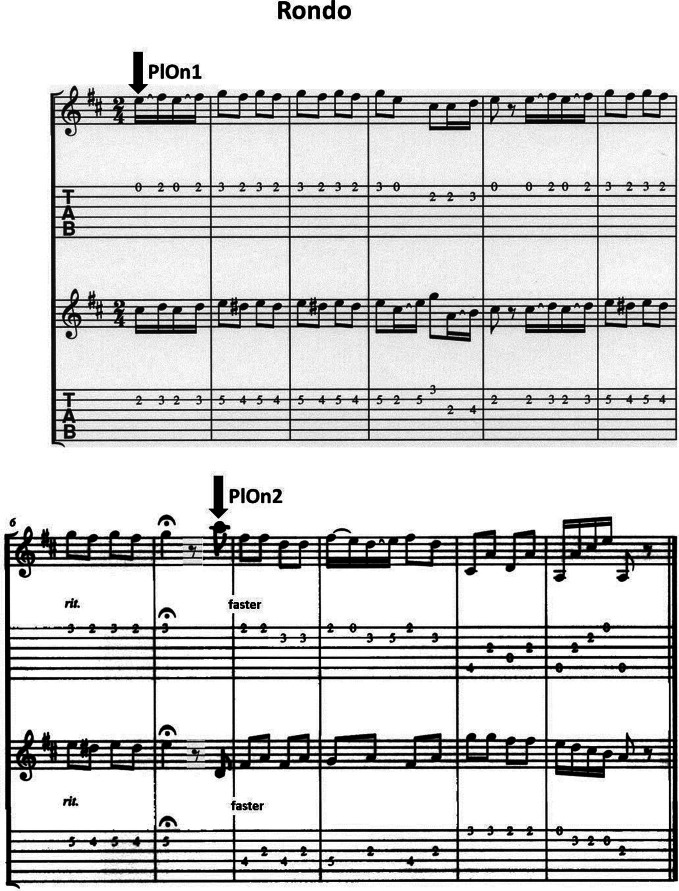
**Music sheet of the adapted version of the Rondo in D Major by C.G. Scheidler; segments of 500 ms before and after the two marked play onsets were analyzed**.

The duets were measured sitting face-to-face to each other in an electromagnetically shielded cabin. During the measurement, they played a short excerpt of a Rondo taken from the Sonata in D Major by Christian Gottlieb Scheidler (1752–1815). The piece was slightly modified to make the two voices musically equivalent. Apart from the initial play onset, the piece featured a decrease in musical tempo (i.e., *ritardando*), an eighth rest, and a subsequent second play onset as an additional coordination point (see Figure 1 for the music sheet). The designated leader was requested to bring the other one in at play onsets, and to manage the playing tempo. The guitarist asked to follow was supposed to comply with the leader. All guitarists played the Rondo sequence by heart. It was played at least 60 times, in two blocks of ~30 trials each. Trials were initiated by four metronome beats (80 bpm), after the last of which the leader cued the follower by calmly breathing in. To minimize movement artifacts, participants were asked to execute small picking movements and to avoid any kind of body movement. For each duet, the measurement was repeated on another day with reversed role assignments to avoid confounding of the aimed-at effects with person variables.

### EEG data acquisition and preprocessing

Sixty-four active Ag/AgCl electrodes (actiCAP, Brain Products, Munich, Germany) were placed on each head according to the international 10–10 system, with the reference electrode at the right mastoid. For each participant, separate amplifiers (BrainAmp DC, BrainProducts, Munich, Germany) with separate grounds were used, however, these were linked to the same computer. Eye blinks and eye movements were controlled by recordings of vertical and horizontal electrooculograms (EOGs). Via two microphones, the guitars were recorded on two separate channels simultaneous to the EEG. Hand movements were tracked by acceleration sensors. For these purposes, a bipolar amplifier was used (BrainAmp ExG, Brain Products, Munich, Germany). All channels were recorded at a sampling rate of 5000 Hz with a bandpass filter of 0.01–1000 Hz. An audio-video recording of each session was also captured in synchrony with the EEG data acquisition (Video Recorder Software, Brain Products, Munich, Germany). Markers for the metronome beats were automatically set online during the measurement, whereas event markers for the two play onsets of the Rondo had to be added offline afterwards, with the help of the audio, video, and movement recordings. EEG data were re-referenced to an average of the left and right mastoid, resampled at 1000 Hz and filtered with a band pass ranging from 1 to 70 Hz. Eye movement correction was accomplished by independent component analysis (Vigário, 1997; Jung et al., 1998). Artifacts from head and body movements were rejected by visual inspection only, after an artifact rejection based on a gradient (a maximum admissible voltage step of 50 μV), and a difference criterion (a maximum admissible absolute difference between two values in a segment of 200 μV) had not rendered satisfactory results. Spontaneous EEG activity was segmented into epochs of 3 s, starting 1 s before the first respectively second play onset of the leading guitarist and ending 2 s after it.

### Definition of frequencies and time segments of interest

Within the alpha and beta bands, we selected “frequencies of interest” (FOIs), based on observations reported by Lindenberger et al. (2009), according to which synchronization maxima are likely to occur at multiples of the metronome frequency. In our study, this was a rhythm of 80 bpm, corresponding to 1.33 Hz. Accordingly, 8, 9.33, 10.67, and 12 Hz were multiples within the alpha band; 13.33, 14.67, 16, 17.33 […], 24, 25.33, 26.67, and 28 Hz were multiples within the beta band. Given that the time frequency analysis, on the basis of which the phase coupling was calculated (see below), had a frequency resolution of 0.5 Hz, we narrowed the selection down to the respective integer values, i.e., 8 and 12 in alpha, 16, 20, 24, and 28 in beta. To reach the same number of FOIs in both bands, we furthermore confined ourselves to 20 and 28 Hz in beta, such that we would have one FOI representative of the lower respectively higher frequencies in both bands. With regard to time, we chose to analyze segments of 500 ms before and after the coordinated play onsets (for details, see Sänger et al., 2012).

### Calculation of directional couplings

We selected 21 electrodes per person (Fp1, Fpz, Fp2, F7, F3, Fz, F4, F8, T7, C3, Cz, C4, T8, P7, P3, Pz, P4, P8, O1, Oz, O2). With these, the entire cortex was still covered but the possibility of overestimating functional connectivity due to volume conductance was reduced.

The subsequent time-frequency and coherence analyses were performed with a LabView-based tool developed by Müller and Lindenberger (2011; LabView, National Instruments, Austin, Texas, USA).

The EEG time series of these channels were transformed into complex time-frequency signals using a complex mother Morlet wavelet, also called Gabor wavelet. The wavelet coefficients were calculated with a time step of 5 leading to a temporal resolution of 5 ms and frequency resolution of 0.5 Hz. From this, the instantaneous phase difference Δϕ_*mn*_ (*t, f*) was calculated between all possible 1764 electrode pairs of each duet in the 500 ms before and after the two play onsets and for the FOIs specified above.

We then derived an asymmetric index of in-phase synchronization between each of two electrode time series: First, points at which the phase angle between the two oscillators ranged between −π/4 and +π/4 were counted, and secondly, this counting was repeated for the positive range only (between 0 and +π/4). To exclude points of incidental coupling, in-phase synchronized points were only counted if they were not accompanied by unsynchronized points within a time window shorter than one oscillation period of the given frequency. Both numbers were then weighted relative to the number of all data points in the time window of 500 ms. Finally, the Absolute Coupling Index (*ACI*), that is, the relative number of all phase-locked points, and the Positive Coupling Index (*PCI*), that is, the relative number of phase-locked points with an angle between 0 and +π/4, were combined in the (*ICI*) by the following formula (Müller and Lindenberger, 2011):

ICI=((PCI+ACI)/(2×ACI))×sqrt(PCI)

where *ICI* is equal to 1 when all points are phase-locked with a positive angle and equal to 0 when all points are phase-locked with a negative phase angle or a phase angle beyond the range of −π/4 to +π/4. *ICI* is an asymmetric measure (*ICI*_AB_ ≠ *ICI*_BA_) and thus indicative of the anteriority of one time series with regard to another. In the following, the ICI_AB_, for example, will thus be interpreted as a quantification of directed coupling from A to B. The coupling was calculated for each trial and then averaged across trials before further analysis (for a more extensive description of these measures see Müller and Lindenberger, 2011).

### Graph analysis of hyperbrain networks

**Figure 2:**
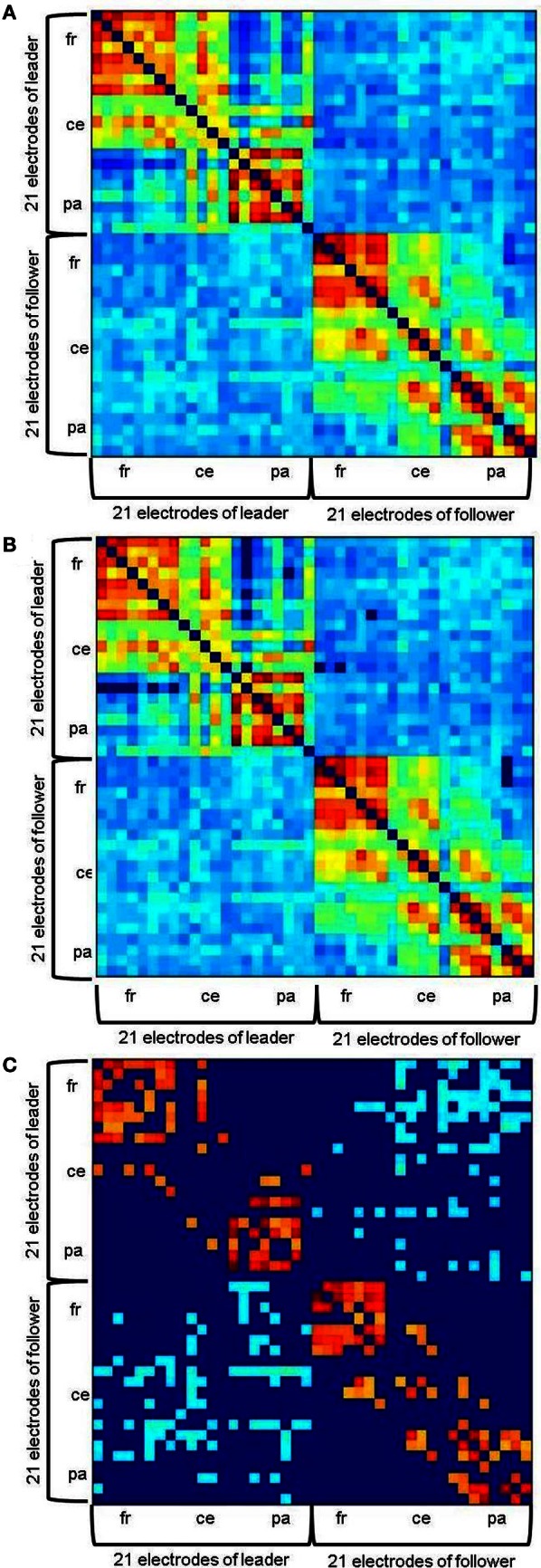
**Example of a hyperbrain network before the first play onset at 20 Hz. (A)** without threshold, **(B)** with an absolute threshold of 0.1102, **(C)** with an additional proportional threshold of 20% applied separately for within- and between brain connections. Within-brain coherence of the leader is captured in the upper left, within-brain coherence of the follower in the lower right. Between-brain coherence is shown in the upper right and lower left of the matrix. The auto-coherence on the main diagonal is set to zero. For each interaction partner, 21 electrodes are arranged in the following order: Fp1, Fpz, Fp2, F7, F3, Fz, F4, F8, T7, C3, Cz, C4, T8, P7, P3, Pz, P4, P8, O1, Oz, O2 from top (leader) to bottom (follower) and left (leader) to right (follower).

The *ICI* values for the 1764 electrode pairs were fed into a graph analysis of hyperbrain networks containing 21 electrodes of both duet partners and accordingly within-brain as well as between-brain couplings (see Figure 2A for an example). Hyperbrain networks were analyzed separately for the segments of 500 ms each before and after the two play onsets as well as for the FOIs of 8, 12, 20, and 28 Hz. The graph analysis was performed using the Brain Connectivity Toolbox developed by Rubinov and Sporns (2010).

#### Thresholding

Thresholding was applied in two steps. To ensure that only significant connections would be evaluated, we firstly determined an absolute significance threshold by means of surrogate data: *ICI* was calculated for shuffled EEG time series and 1000 bootstrapping samples were drawn from the resulting values. The absolute threshold was then defined as *M*_boot_ + 3 × *SD*_boot_. This resulted in the critical values of 0.1102 for 8 Hz, 0.1340 for 12 Hz, 0.1690 for 20 Hz, and 0.1901 for 28 Hz. *ICI* values lower than these thresholds were accordingly set to zero in the hyperbrain networks (Figure 2B). Second, a proportional threshold was applied such that only the same number of strongest couplings remained in each network. It is important to note, however, that this was done separately for the within- and the between-brain part of the networks. Absolute values are of course substantially higher for within- than for between-brain coupling, meaning that between-brain coupling would probably be entirely eliminated if such a procedure was applied for both types of coupling together. To find an appropriate thresholding proportion that would uncover a meaningful network topography, we took the networks' small-worldness as a criterion. Small-world networks are characterized by an optimal balance of functional integration and segregation (cf. Rubinov and Sporns, 2010). According to Watts and Strogatz, this is given if a network is more clustered than a random network, but shows approximately the same characteristic path length (Watts and Strogatz, 1998). These relations were reliably reached in the considered hyperbrain networks when applying a threshold of 20%. Thus, the networks we finally analyzed contained only the strongest 20% of their original within- and between-brain couplings, respectively (Figure 2C). For a detailed description of the analysis of the hyperbrain networks' small-worldness, see **Appendix B**.

#### Calculation of connection strengths

To quantify the coupling in the hyperbrain networks, we chose the measure of out-strengths (*OS*), which yields, for each network node, the sum of weighted links emanating from this node to all other nodes in the network (Rubinov and Sporns, 2010). In the context of our coupling measures then, one electrode's *OS* is the sum of the respective *ICI* values that link this electrode to any other electrode in the network. After calculating *OS* for each electrode separately, we averaged them for three broader cortical sites: prefrontal-frontal, containing Fp1, Fpz, Fp2, F7, F3, Fz, F4, and F8, central-temporal, containing T7, C3, Cz, C4, T8, and parietal-occipital with P7, P3, Pz, P4, P8, O1, Oz, and O2. In the following, these three sites are conveniently referred to as “frontal,” “central,” and, respectively, “parietal.” We calculated the *OS* for the entire hyperbrain network by summing up the *ICI* values across all other 41 electrodes in the network. Also, we calculated OS for the within-brain shares corresponding to the leader, respectively follower only by summing up across the other 20 electrodes of the same brain. By subtracting these within-brain out-strengths (*wOS*) from the hyperbrain out-strengths (*hOS*), we also captured the between-brain out-strengths (*bOS*), which refer to couplings between brains only and are the most interesting measure with regard to our hypotheses.

#### Statistical evaluation of strengths

For *hOS* and *bOS*, we calculated five-way repeated measures ANOVAs in the alpha and beta bands, respectively. In these, we considered the factors BAND (low alpha, respectively beta vs. high alpha, respectively beta), PLAY ONSET (first vs. second), TIME (before vs. after the respective play onset), COUPLING DIRECTION (leader to follower versus follower to leader), and SITE (frontal vs. central vs. parietal). The only difference in the ANOVA-based evaluation of *wOS* was that the factor COUPLING DIRECTION was replaced by the factor ROLE, with its levels being within leader vs. within follower. The alpha level was set to *p* = 0.05. Statistically significant effects were followed up by *post-hoc* ANOVAs or pairwise comparisons. The values for significant *post-hoc* tests are summarized in **Appendix C**. Greenhouse–Geisser epsilons were used for non-sphericity correction where necessary.

## Results

### Couplings in the alpha range

#### Directed out-strengths in hyperbrain networks

For *hOS*, we found main effects of BAND [*F*_(1, 23)_ = 184.33, *p* < 0.001, η^2^_*p*_ = 0.60] and SITE [*F*_(2, 46)_ = 40.42, *p* < 0.001, η^2^_*p*_ = 0.64]. hOS were stronger at 12 than at 8 Hz and stronger at frontal and central than at parietal sites (Table C1). These main effects were qualified by an interaction of BAND and SITE [*F*_(2, 46)_ = 6.66, *p* < 0.005, η^2^_*p*_ = 0.23], which showed that the difference between coupling at 8 vs. 12 Hz did not apply to frontal sites (Table C2). We furthermore found an interaction of SITE and PLAY ONSET [*F*_(2, 46)_ = 3.97, *p* < 0.05, η^2^_*p*_ = 0.15]: Frontal *hOS* were stronger at the first play onset, while central *hOS* were higher at the second play onset (Table C3).

#### Directed out-strengths in within-brain networks

Effects for *wOS* were virtually the same as those for *hOS* and are therefore summarized in Tables C2–C4.

#### Directed out-strengths in between-brain networks

**Figure 3:**
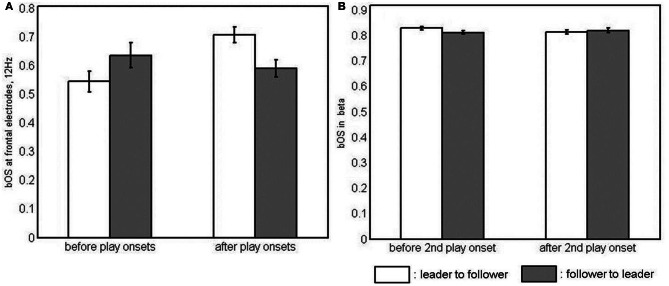
**Interaction of coupling direction and time in simple-effects ANOVAs of between-brain out-strengths (*bOS*). (A)** at frontal electrodes at 12 Hz and **(B)** in the beta band, i.e., collapsed across 20 and 28 Hz and all electrode sites, as neither the frequency band nor the electrode site had an effect here.

There were main effects of BAND [*F*_(1, 23)_ = 322.90, *p* < 0.001, η^2^_*p*_ = 0.93] and SITE [*F*_(1.45, 33.42)_ = 4.14, *p* < 0.05, η^2^_*p*_ = 0.15] for *bOS.* They were generally stronger at 12 than at 8 Hz and stronger at parietal than at frontal sites (*t* = −2.46, *p* < 0.05). In the interaction of the two factors [*F*_(1.58, 36.38)_ = 6.12, *p* < 0.01, η^2^_*p*_ = 0.21], however, differences between sites held true for 12 Hz only. Here, parietal electrodes were more strongly involved than frontal and central electrodes (Table C2). Given a four-way interaction of BAND, COUPLING DIRECTION, TIME, and SITE [*F*_(2, 46)_ = 3.48, *p* < 0.05, η^2^_*p*_ = 0.13], we collapsed across the two play onsets and calculated *post-hoc* ANOVAS for COUPLING DIRECTION, TIME, and SITE at both levels of BAND, namely, 8 and 12 Hz. We did not find effects at 8 Hz. At 12 Hz, however, there was an interaction of COUPLING DIRECTION, TIME, and SITE [*F*_(1.57, 36.15)_ = 6.07, *p* < 0.01, η^2^_*p*_ = 0.21], which is illustrated in Figure 3A. To break this interaction down further, we again calculated *post-hoc* ANOVAs, this time for all levels of SITE, and found an interaction of COUPLING DIRECTION and TIME at frontal [*F*_(1, 23)_ = 9.04, *p* < 0.01, η^2^_*p*_ = 0.28] and parietal electrodes [*F*_(1, 23)_ = 6.72, *p* < 0.05, η^2^_*p*_ = 0.23]. *Post-hoc* comparisons revealed that only leaders' frontal *bOS* increased after the play onsets, resulting in a reliable dominance of leaders' vs. followers' frontal *bOS* after the play onsets (Table C5). Leaders' parietal *bOS* decreased after the play onsets. This did not result in a reliable leader-follower difference, however.

### Couplings in the beta range

#### Directed out-strengths in hyperbrain networks

There were main effects of TIME [*F*_(1, 23)_ = 5.46, *p* < 0.05, η^2^_*p*_ = 0.19] and BAND [*F*_(1, 23)_ = 65.32, *p* < 0.001, η^2^_*p*_ = 0.74], indicating stronger hOS for the time segments after vs. before the play onsets, and stronger *hOS* for 28 vs. 20 Hz. This main effect of BAND was qualified by an interaction with SITE [*F*_(2, 46)_ = 11.89, *p* < 0.001, η^2^_*p*_ = 0.34]: The significant difference between the two beta frequencies applied only to central and parietal electrodes. At frontal electrodes, there was no difference between the two beta frequencies. At 20 Hz, *hOS* were highest at frontal electrodes (Table C2). The interaction of PLAY ONSET, COUPLING DIRECTION, and TIME [*F*_(1, 23)_ = 7.50, *p* < 0.05, η^2^_*p*_ = 0.25] was broken down to *post-hoc* ANOVAs of COUPLING DIRECTION and TIME at both play onsets, but except for a main effect of time at the first play onset these failed to render significant effects.

#### Directed out-strengths in within-brain networks

Main effects of PLAY ONSET [*F*_(1, 23)_ = 8.08, *p* = 0.01, η^2^_*p*_ = 0.26] and TIME [*F*_(1, 23)_ = 12.96, *p* = 0.01, η^2^_*p*_ = 0.36] indicated that *wOS* were stronger at the second than at the first play onset and stronger after the play onsets than before. Furthermore, there was a significant interaction of BAND and SITE [*F*_(2, 46)_ = 27.26, *p* < 0.001, η^2^_*p*_ = 0.54]. At 20 Hz, frontal *wOS* were stronger than *wOS* at the other sites. At frontal sites, *wOS* were stronger at 20 than at 28 Hz, while at parietal sites, *wOS* were stronger at 28 than at 20 Hz (Table C2).

#### Directed out-strengths in between-brain networks

Apart from a main effect of BAND [*F*_(1, 23)_ = 161.75, *p* < 0.001, η^2^_*p*_ = 0.88], indicating stronger *bOS* for 28 than for 20 Hz, we found a three-way interaction of PLAY ONSET, TIME, and COUPLING DIRECTION [*F*_(1, 23)_ = 8.58, *p* < 0.01, η^2^_*p*_ = 0.27]. A *post-hoc* ANOVA of COUPLING DIRECTION and TIME, collapsed across frequencies and sites, did not show significant effects for the first play onset. For the second play onset, however, there was a significant interaction of COUPLING DIRECTION and TIME [*F*_(1, 23)_ = 6.72, *p* < 0.05, η^2^_*p*_ = 0.23], which is depicted in Figure 3B. *Post-hoc* comparisons revealed that *bOS* of leaders were higher than those of followers' before, but not after the second play onset, as they significantly decreased from before to after the play onset (Table C6).

## Discussion

### Summary and interpretation of findings

Taking on different roles is an important aspect of action coordination between individuals. Interaction partners coordinate their actions with each other regardless of whether their individual contributions to the joint action are identical, similar, or different. We expected that this coordination process would be reflected in the presence of hyperbrain networks with directed neural couplings. To test this assumption, we applied a measure of in-phase synchronization, namely the ICI (Müller and Lindenberger, 2011), to the EEG series of two guitarists playing a short Rondo sequence by C. G. Scheidler. The ICI indicates the direction of the coupling in the sense that it shows the relative degree of anteriority of one time-series over another, or, in other words, the time-lagged synchronization between two signals. Major results are summarized and discussed below.

#### Findings regarding directed couplings in the alpha range

Findings were identical for hyperbrain and within-brain networks, pointing to a dominance of within-brain coupling in the hyperbrain networks, presumably reflecting a confluence of functional and anatomical connectivity for nodes located in the same brain. This led us to interpret these findings in the within-brain realm only, in which *OS* can be interpreted as an indicator of activation (cf. Astolfi et al., 2010).

We found that frontal sites were generally more active than other cortical sites. Although associations between neuroelectrical and imaging studies should be treated with caution due to their methodological differences, it can be said that this finding is consistent with prior results relating frontal sites with functions that underlie IAC (e.g., McCabe et al., 2001; Decety et al., 2002, 2004). Besides executive functions, which certainly were of concern here, frontal areas have been linked to auditory-motor mapping, an important process in music production (Bangert and Altenmüller, 2003; Sauseng et al., 2005). Furthermore, we saw that the activation of frontal sites was higher around the first play onset, while central sites were more active around the second play onset. This might be related to the role of the prefrontal cortex in action planning (e.g., Mushiake et al., 2006), a function that is likely to be more prominent at the very beginning of a piece than in its middle, when playing is underway. Higher activation of central electrodes around the second play onset might hint at stronger mirroring processes taking place at this point in time (cf. Oberman et al., 2007), not to mention activation of premotor and motor areas during guitar playing. For the second play onset, there was no external orienting stimulus as there was for the first play onset. Although the metronome was not supposed to trigger the initial joint onset, it may still have served as a signal. At the second onset, though, the players had to rely exclusively on their own coordinative functions. It is thus conceivable that mirror neuron activity was more in demand at this point of the piece.

With regard to our hypotheses, findings for the between-brain parts of the hyperbrain networks were most important: We saw that parietal electrodes were more strongly involved in between-brain coupling than other cortical sites. This is in line with several prior results, which have shown a clear involvement of parietal regions in between-brain associations during interaction (Anders et al., 2010; Astolfi et al., 2010; Dumas et al., 2010; Stephens et al., 2010), amongst others our own finding that parietal nodes form part of modules of strong interbrain connectivity as measured with trial-based, symmetric coherence measures (Sänger et al., 2012). Most interestingly, though, there were significant effects in association with the coupling direction: Only the strength of coupling going from the leader to the follower changed significantly from before to after the play onsets. Specifically, coupling going from the leader's frontal nodes to the follower's brain at 12 Hz was significantly higher after than before the play onsets, whereas coupling going from the leader's parietal nodes to the follower's brain was significantly lower after the play onsets. The increase of coupling emanating from the leader's frontal nodes resulted in frontal leader-to-follower coupling being significantly stronger than frontal follower-to-leader coupling after the play onsets.

Hence, as predicted, we observed some evidence indicating that asymmetries in between-brain couplings might reflect differences between leaders and followers during IAC. Apparently, processes coded in a high alpha frequency at frontal sites were coupled in a time-delayed fashion between the leader's and the follower's brain after the play onsets. The follower was instructed to exclusively orientate himself toward the leader, that is, to react to what he or she is doing. Hence, the observed asymmetry in coupling parameters may point to interpersonally coordinated, time-lagged action-monitoring processes in the follower (cf. Ridderinkhof et al., 2004). Put more generally, the observed localization of leader-follower asymmetries at frontal and parietal sites points to mechanisms related to temporal aspects of action-perception integration over time, a function that is likely to matter in joint music production (cf. Quintana and Fuster, 1999). An association with the alpha-based sensori-fronto-parietal network can be assumed, which is supposed to mediate the bringing to awareness of sensory information (Palva and Palva, 2007). Furthermore, focusing on the alpha band, it is interesting to consider that it has been attributed a direct role in attention and the processing of internal tasks (Palva and Palva, 2007), which can be related to the fact that the guitarists played the Rondo by heart. Also its potential timing function might give alpha a central role in the time-sensitive process of making music (Klimesch et al., 2007).

#### Findings regarding directed coupling in the beta range

As was true for alpha, the hyperbrain results for beta were apparently dominated by what could be observed at the level of within-brain networks. Again, frontal sites were more strongly involved than other cortical sites. Taking into account again that the guitarists played by heart, this could be related to a possible role of frontal beta in memory rehearsing processes (Tallon-Baudry et al., 1999). Additionally, we found generally stronger directed coupling after than before the play onsets, which might reflect the onset of motor processes in both players (cf. Pfurtscheller, 1981; Pfurtscheller et al., 1996). For beta frequencies, the effect of coupling direction did not depend on cortical site. Before the second play onset, the leader-to-follower coupling was stronger than the follower-to-leader coupling. It decreased, however, in the segment after the play onset, such that the difference between directions was no longer reliable. Thus, a second indication of asymmetries in between-brain couplings as a function of role was found. In relation to the second onset of play, we may tentatively conclude that processes in the beta range were asymmetrically coupled between the two brains before the actual onset of playing. Perhaps this reflects anticipatory imagery of finger movements (Schnitzler et al., 1997). Why this effect was found only before the second and not before the first play onset, is not easily understood. Activity triggered by the metronome beats may have overshadowed activity related to imagined finger movement.

### Limitations and open questions

Research on the brain basis of IAC is still in its beginnings. Looking for effects in between-person space cannot yet rely on a set of well-established methods and procedures. Hence, the present findings should be interpreted with some caution, and may need to be reconsidered in the light of further experimental paradigms and data-analytic innovations. Thus, the validity of the present results may be limited by a number of factors, some of which seem particularly important to address.

First, the present analyses were restricted to time segments of 500 ms surrounding the coordinated play onsets only. Arguably, coordinated play onsets are points in time that place special demands in IAC (Sänger et al., 2012). Nevertheless, some of the mechanisms related to phase-coupling may require a larger time window to be properly assessed. This consideration is less of a concern for alpha and beta frequencies but identifying the contribution of lower frequencies to IAC would require larger time windows.

Second, it would be desirable to analyze inter-brain couplings in relation to different musical events, and in relation to variations in behavior. For instance, in the musical piece used in the current study, interesting points were not only the play onsets, but, for example, the ritardando (decrease of tempo) and the fermata (hold) before the rest, as well as the accelerando (increase of tempo) after the rest. Unfortunately, the audio recording we took from each guitar was not accurate enough to unambiguously identify the individual notes indicating these events. Future studies may make use of synchronized midi files, which allow setting markers denoting the onset of each single note in the EEG data, to overcome this limitation. This would also aid attempts to find inter-brain and intra-brain signatures of behavioral coordination success. In this context, it could furthermore be interesting to take into account the phenomenon that skillful deviations from precise timing, instead of strict adherence to tempo, are at times desirable (Keil and Feld, 1994; Janata et al., 2012).

Third, the investigation of cross-frequency (n:m) neural couplings during IAC will be essential in the future, especially for the specific example of jointly playing music, in which the coordination of different time scales can be an important aspect.

Finally, it should be mentioned that movement artifacts cannot be fully eliminated or controlled in a natural IAC setting such as guitar dueting. As mentioned earlier, we tried to minimize such artifacts by instructing the participants to keep their movements as small as possible, and of course, an EEG artifact rejection was performed. In this context, we acknowledge that time-delayed motor potentials in association with slight, unintended asynchronies between the respective play onsets of both players cannot fully be excluded as an explanatory factor for the directed couplings we observed between the leaders' and the followers' brains. We would like to point out, however, that our major findings, namely the dominance of leader-to-follower coupling in alpha, at frontal electrodes, after the play onsets, and in beta, before the second play onset do not suggest a crucial role of the addressed movement-related potentials when considering the combination of frequency, topology, and timing in the results.

## Conclusion

Using duet guitar playing as a paradigm, we identified coupled activity in the alpha and beta frequency ranges within and between two brains. Notably, and as predicted, the directionality of the observed couplings varied as a function of the musical roles of leader and follower. The cortical sites and frequencies of the observed leader-follower differences were in line with available evidence on cortical mechanisms supporting IAC. In addition, leader-to-follower couplings tended to be more pronounced than follower-to-leader couplings, presumably pointing to the greater causal influence of the leader in IAC on the temporal parameters of joint action.

In sum, we were able to show that role differences between two human agents engaged in coordinated action are reflected in the directionality of oscillatory couplings between their brains. We conclude that functional hyperbrain networks, construed by indices of directed connectivity within and between brains, appear to be a promising tool to explore and delineate neural mechanisms of IAC.

### Conflict of interest statement

The authors declare that the research was conducted in the absence of any commercial or financial relationships that could be construed as a potential conflict of interest.

## Figures and Tables

**Figure B1:**
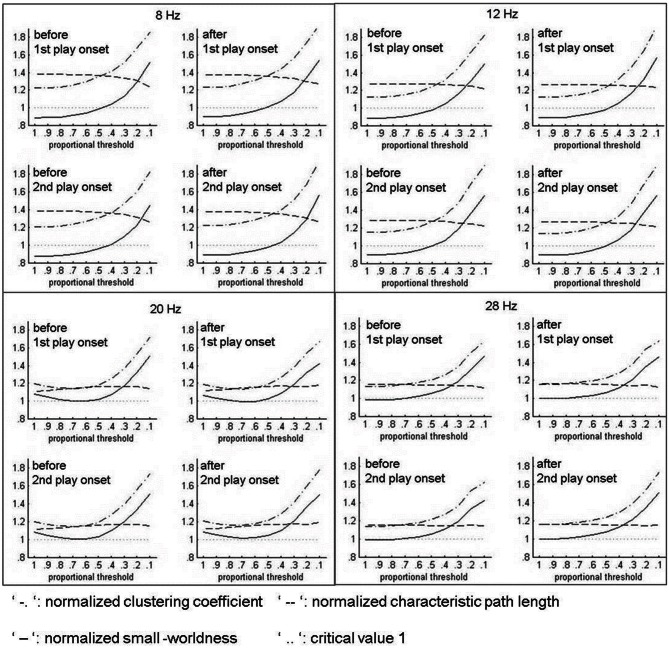
**Indices of normalized small-worldness (*SW*_*norm*_) at different proportional thresholds**.

## Appendices

### Appendix A

**Table A1:** **Matching of age and qualification aspects of guitarists A and B in the 12 duets of the final sample**.

	**Age**		**Experience**		**Conservatory**		**Work**		**Ensemble**	
	**A**	**B**	**A**	**B**	**A**	**B**	**A**	**B**	**A**	**B**
1	30	41	12	17	n	n	n	n	y	y
2	46	46	38	39	y	y	y	y	y	y
3	58	50	48	20	n	n	n	n	n	n
4	20	25	11	14	y	y	n	y	y	y
5	29	40	18	30	y	y	y	y	y	y
6	41	47	25	37	n	n	y	y	y	y
7	26	30	10	11	n	n	n	n	n	y
8	30	28	17	15	n	n	y	y	n	n
9	37	28	29	16	y	n	y	y	y	y
10	44	47	30	33	n	n	n	n	y	n
11	29	22	10	7	n	n	n	n	n	n
12	49	20	25	13	y	y	y	n	y	n
	Mean		Mean		11 out of 12		10 out of 12		9 out of 12	
	difference = 7.92		difference = 7.92		concordant		concordant		concordant	
	(*SD* = 7.48)		(*SD* = 7.93)							

Experience, years since participant started to play the guitar.

Conservatory, participant has studied/is studying guitar (y) or not (n).

Work, participant works as guitarist (y) or not (n).

Ensemble, participant is currently a member of a music ensemble (y) or not (n).

### Appendix B

#### Proportional thresholding in hyperbrain networks

In order to uncover a meaningful topography in the hyperbrain networks, it was necessary to find an appropriate thresholding proportion. We therefore examined the networks' small-worldness at different proportional thresholds.

Small-worldness is considered an optimal architecture of functioning networks (Stam, 2004) and stands for an optimal balance of functional integration and segregation (cf. Rubinov and Sporns, 2010). Functional integration is commonly indicated by the Characteristic Path Length (*CPL*) of a network, which represents the average shortest path between two nodes in this network. Functional segregation is generally quantified by the Clustering Coefficient (*CC*), which is the share of a node's neighbors that are also neighbors of each other. Watts and Strogatz (1998) have accordingly characterized small-world networks as being more clustered than random networks, but showing approximately the same characteristic path length as random networks.

This notion is formalized in the index of normalized small-worldness (*SW*_*norm*_), which equals the quotient of normalized Clustering Coefficient (*CC*_*norm*_) and normalized Characteristic Path Length (CPLnorm; Rubinov et al., 2009). These normalized indices of *CC* and *CPL* are determined by dividing their values observed in the networks concerned (*CC*_*obs*_ resp. *CPL*_*obs*_) by their values in random networks (*CC*_*rand*_ resp. *CPL*_*rand*_). We thus built random networks by shuffling the 1764 links in the observed hyperbrain networks and computed *CC*_*rand*_ and *CPL*_*rand*_. In accordance with the specification of small-world networks stated above (Watts and Strogatz, 1998), *CC*_*norm*_ (=*CC*_*obs*_/*CC*_*rand*_) should be greater than 1, while *CPL*_*norm*_ (=*CPL*_*obs*_/*CPL*_*rand*_) should be around 1 (Rubinov et al., 2009). *SW*_*norm*_ (=*CC*_*norms*_/*CPL*_*norm*_) should thus be greater than 1.

We repeated these calculations for 10 different proportional thresholds between 100 and 10%, meaning that 100, 90, 80,…, 20, or respectively 10% of the strongest couplings remained in the network.

As can be seen in the graphs below, *CC*_*norm*_ lay over 1, and therefore in the desired range, for all considered thresholds and time segments. However, it substantially exceeded its initial value only for thresholds that were stricter than 60%. The level of *CPL*_*norm*_, by contrast, lay a little too high when applying the criteria formulated by Watts and Strogatz (1998), and hardly changed with different thresholds. In any case, *CC*_*norm*_ outran *CPL*_*norm*_ for thresholds stricter than 0.5, and accordingly their ratio, *SW*_*norm*_, rose over 1 for these thresholds. Furthermore, *SW*_*norm*_ itself became greater than *CPL*_*norm*_, at least for thresholds of 20 and 10%. As both of these relations are in line with small-worldness (*CC*_*norm*_ > *CPL*_*norm*_ and *SW*_*norm*_ > *CPL*_*norm*_) we selected 20%, the proportion at which these relations are established in any case, as the proportional threshold. Thus, the networks that we finally analyzed contained only the strongest 20% of their original within- and between-brain couplings, respectively.

### Appendix C

Supplementary tables showing means (*M*), standard deviations (*SD*), *t*, and *p*-values of hypothesis-relevant, significant follow-up *t*-tests for the repeated-measures ANOVAs of out-strengths in hyperbrain networks, within-, and between-brain networks.

**Table C1:** **Main effect of SITE for *hOS* in the alpha band**.

	***M***	***SD***		***M***	***SD***	***t***_**(23)**_	***p***
Parietal	1.94	0.06	Frontal	2.85	0.07	8.19	<0.001
			Central	2.01	0.06	7.08	<0.001

**Table C2:** **Interaction of BAND and SITE for *hOS, wOS*, and *bOS* in alpha and beta bands**.

		***M***	***SD***		***M***	***SD***	***t***_**(23)**_	***p***
**ALPHA**								
*hOS*								
8 Hz	Frontal	2.82	0.07	Central	1.93	0.06	9.03	<0.001
				Parietal	1.79	0.06	8.58	<0.001
12 Hz	Frontal	2.88	0.08	Central	2.10	0.07	6.78	<0.001
				Parietal	2.09	0.07	5.43	<0.001
Central	8 Hz	1.93	0.06	12 Hz	2.10	0.07	−3.58	0.001
Parietal	8 Hz	1.79	0.06	12 Hz	1.79	0.06	−7.39	<0.001
*wOS*								
8 Hz	Frontal	2.27	0.07	Central	1.36	0.06	9.23	<0.001
				Parietal	1.23	0.06	8.64	<0.001
12 Hz	Frontal	2.26	0.08	Central	1.45	0.07	7.69	<0.001
				Parietal	1.36	0.07	6.76	<0.001
Central	8 Hz	1.36	0.06	12 Hz	1.45	0.07	−2.39	<0.05
Parietal	8 Hz	1.23	0.06	12 Hz	1.36	0.07	−4.39	<0.001
*bOS*								
12 Hz	Frontal	0.62	0.02	Parietal	0.74	0.02	−3.09	0.005
	Central	0.65	0.02	Parietal	0.74	0.02	−2.30	<0.05
Frontal	8 Hz	0.55	0.01	12 Hz	0.62	0.02	−4.13	<0.001
Central	8 Hz	0.56	0.01	12 Hz	0.65	0.02	−5.45	<0.001
Parietal	8 Hz	0.56	0.02	12 Hz	0.74	0.02	−7.18	<0.001
**BETA**								
*hOS*								
20 Hz	Frontal	2.72	0.09	Central	2.38	0.07	2.77	<0.05
				Parietal	2.38	0.08	2.16	<0.05
Central	20 Hz	2.38	0.07	28 Hz	2.46	0.07	−2.26	<0.05
Parietal	20 Hz	2.38	0.08	28 Hz	2.56	0.09	−6.81	<0.001
*wOS*								
20 Hz	Frontal	1.93	0.09	Central	1.59	0.07	2.90	<0.01
				Parietal	1.58	0.08	2.31	<0.05
Frontal	20 Hz	1.93	0.09	28 Hz	1.80	0.09	6.06	<0.001
Parietal	20 Hz	1.58	0.08	28 Hz	1.74	0.09	6.59	<0.001

**Table C3:** **Interaction of SITE and PLAY ONSET (PlOn) for *hOS* and *wOS* in alpha**.

		***M***	***SD***		***M***	***SD***	***t***_**(23)**_	***p***
*hOS*								
PlOn 1	Frontal	2.89	0.08	Central	1.98	0.06	8.57	<0.001
				Parietal	1.93	0.06	7.27	<0.001
PlOn 2	Frontal	2.80	0.07	Central	2.05	0.07	7.28	<0.001
				Parietal	1.96	0.07	6.31	<0.001
Frontal	PlOn 1	2.89	0.08	PlOn 2	2.80	0.07	2.35	<0.05
Central	PlOn 1	1.98	0.06	PlOn 2	2.05	0.07	−2.33	<0.05
*wOS*								
PlOn 1	Frontal	2.29	0.08	Central	1.38	0.06	8.93	<0.001
				Parietal	1.28	0.06	8.07	<0.001
PlOn 2	Frontal	2.26	0.07	Central	1.44	0.06	8.10	<0.001
				Parietal	1.71	0.06	7.23	<0.001
Frontal	PlOn 1	2.29	0.08	PlOn 2	2.26	0.07	2.27	<0.05
Central	PlOn 1	1.38	0.06	PlOn 2	1.44	0.06	−2.46	<0.05

**Table C4:** **Main effects for *wOS* in alpha**.

BAND [*F*_(1, 23)_ = 56.70, *p* < 0.001, η^2^_*p*_ = 0.71]							
SITE [*F*_(2, 46)_ = 48.53, *p* < 0.001, η^2^_*p*_ = 0.68]							
	***M***	***SD***		***M***	***SD***	***t***_**(23)**_	***p***
Frontal	2.26	0.07	Central	1.41	0.06	8.75	<0.001
			Parietal	1.29	0.06	7.84	<0.001

**Table C5:** **Interaction of DIRECTION and TIME for *bOS* at 12 Hz, collapsed across both play onsets**.

	***M***	***SD***		***M***	***SD***	***t***_**(23)**_	***p***
**FRONTAL**							
Leader to follower							
Before	0.55	0.04	After	0.71	0.03	–3.60	<0.01
After							
Leader to follower	0.71	0.03	Follower to leader	0.59	0.03	2.86	<0.01
**PARIETAL**							
Leader to follower							
Before	0.81	0.04	After	0.65	0.03	3.72	<0.001

**Table C6:** **Interaction of DIRECTION and TIME for *bOS* in beta at 2nd play onset, collapsed across both bands and all electrode sites**.

	***M***	***SD***		***M***	***SD***	***t***_**(23)**_	***p***
**LEADER TO FOLLOWER**							
Before	0.83	0.01	After	0.81	0.01	2.21	<0.05
**BEFORE**							
Leader to follower	0.83	0.01	Follower to leader	0.81	0.01	3.02	<0.01
